# A Robust Deep Learning Approach for COPD Automated Detection

**DOI:** 10.3390/s26092713

**Published:** 2026-04-28

**Authors:** Shuting Xu, Ravinesh C. Deo, Salvin S. Prasad, Prabal D. Barua, Jeffrey Soar, Rajendra Acharya

**Affiliations:** 1 Artificial Intelligence Applications Laboratory, School of Mathematics, Physics and Computing, University of Southern Queensland, Springfield, QLD 4300, Australia; shuting.xu@unisq.edu.au (S.X.); prabal.barua@unisq.edu.au (P.D.B.);; 2 Cogninet AI, Sydney, NSW 2010, Australia; 3 School of Sciences, College of Engineering, and Technical Vocational Education and Training, Fiji National University, Samabula, Suva 3722, Fiji; salvin.prasad@fnu.ac.fj; 4 School of Business, University of Southern Queensland, Springfield, QLD 4350, Australia; jeffrey.soar@unisq.edu.au

**Keywords:** asthma detection, random forest, respiratory sound, spectrogram

## Abstract

COPD remains a prevalent and debilitating respiratory condition, necessitating early and accurate diagnosis for optimal clinical intervention. In this study, we propose a novel deep learning-based diagnostic framework that employs the ECAPA-TDNN (Emphasized Channel Attention, Propagation and Aggregation—Time Delay Neural Network) architecture to classify respiratory sound signals from the ICBHI dataset. Originally designed for speaker verification, ECAPA-TDNN introduces channel attention and multi-scale feature aggregation, which we adapt for the first time to the domain of medical acoustic analysis. This architecture allows the model to effectively capture subtle and discriminative patterns in pathological breathing sounds, overcoming the limitations of conventional CNN-based methods. Our methodology integrates rigorous signal preprocessing, log-Mel spectrogram extraction, and data augmentation to enhance robustness and generalization. An Attentive Statistics Pooling mechanism is employed for temporal feature summarization, while Grad-CAM-based explainability is incorporated to improve the interpretability of the diagnostic predictions. The model is rigorously validated using a five-fold cross-validation scheme, achieving a mean validation accuracy of 96.8% with consistently high F1-scores and recall rates across all folds. Comparative analysis with prior methods highlights the superiority of our ECAPA-TDNN-based approach in terms of diagnostic precision, robustness, and potential clinical applicability. To the best of our knowledge, this is the first work to adapt ECAPA-TDNN for COPD detection from respiratory sounds, establishing a new benchmark in interpretable and high-performance acoustic-based respiratory disease screening.

## 1. Introduction

Chronic Obstructive Pulmonary Disease (COPD) is a leading cause of morbidity and mortality worldwide, affecting millions and placing a significant burden on healthcare systems. Early and accurate detection is critical for managing COPD progression and reducing hospitalizations. Conventional diagnostic methods, such as spirometry and clinical auscultation, although widely accepted, are resource-intensive, operator-dependent, and often unavailable in low-resource settings. This highlights the urgent need for alternative, accessible diagnostic tools [1].

In recent years, Artificial Intelligence (AI) has emerged as a transformative technology in healthcare, offering novel approaches for disease detection through pattern recognition and data-driven decision-making. In the context of respiratory diseases, prior studies have demonstrated the effectiveness of Machine Learning (ML) and deep learning techniques in analyzing respiratory sound signals. For instance, Convolutional Neural Network (CNN) and Support Vector Machine (SVM) have been widely applied to classify abnormal lung sounds and detect respiratory conditions [2]. These acoustic signals are rich in diagnostic information, capturing airflow obstruction as well as abnormal breath sounds such as wheezes and crackles, which are hallmark features of COPD. Deep Learning (DL) approaches, in particular, have shown superior performance by automatically learning hierarchical feature representations from raw audio data, reducing the reliance on handcrafted features. This study leverages the Emphasized Channel Attention, Propagation and Aggregation Time Delay Neural Network (ECAPA-TDNN) architecture—a DL model originally designed for speaker verification—to classify COPD and healthy cases using respiratory sounds from the Kaggle Respiratory Sound Database. Compared with conventional DL architectures, such as CNN and Recurrent Neural Networks (RNN), ECAPA-TDNN provides enhanced capability in modeling complex temporal and channel-wise dependencies in respiratory sound signals. CNN-based models primarily focus on local spatial patterns in spectrogram representations but often struggle to capture long-range temporal dependencies inherent in breathing cycles. Similarly, RNN-based models, although effective in sequence modeling, are computationally expensive and prone to vanishing gradient issues when handling long audio sequences [3].

More recently, transformer-based models have shown promising performance in audio classification tasks due to their global attention mechanisms. However, they typically require large-scale datasets and high computational resources, which may limit their applicability in medical audio analysis where data availability is often constrained.

In contrast, ECAPA-TDNN integrates SE-Res2Blocks and attention-based pooling mechanisms, enabling efficient multi-scale temporal modeling and channel-wise feature recalibration. This allows the model to capture both local acoustic variations (e.g., wheezes and crackles) and global temporal structures within respiratory cycles more effectively. Furthermore, its relatively lightweight architecture makes it well-suited for medical applications with limited data and computational constraints [4]. Despite these advancements, translating AI-based respiratory sound classifiers into real-world clinical workflows involves several practical and regulatory challenges. First, performance may degrade under uncontrolled acquisition conditions (e.g., diverse microphones, background noise, different patient postures, and variations in breathing effort), which can introduce domain shift and calibration drift. Second, deployment requires clearly defined intended use (screening vs. diagnostic support), well-specified operating thresholds, and robust quality-control mechanisms to flag low-quality recordings and out-of-distribution inputs. Third, clinical adoption depends on interpretability, human-in-the-loop verification, and workflow integration to minimize false alarms and unnecessary referrals. Finally, regulatory compliance and governance must be considered, including patient consent, privacy-preserving data handling, bias and fairness assessment across demographic subgroups, and prospective validation in representative clinical settings. Moreover, sustainable implementation will require standardized recording protocols, continuous post-deployment monitoring, and periodic model updates to address performance drift and evolving clinical requirements. Therefore, future work will focus on external and prospective validation across multiple sites/devices, robustness and calibration strategies for real-world audio variability, and documentation aligned with medical AI regulatory expectations to support safe and reproducible deployment.

### 1.1. Background and Objectives

COPD is a prevalent respiratory disorder characterized by persistent airflow limitation and progressive airway inflammation [5]. Although spirometry remains the clinical gold standard for diagnosis, its reliance on specialized equipment and trained personnel limits its use in early screening and primary-care settings. Respiratory sounds provide a convenient and non-invasive alternative, as abnormal patterns such as wheezes and crackles often reflect airway obstruction and structural changes in COPD patients [6].

Traditional handcrafted features, such as Mel-Frequency Cepstral Coefficients (MFCCs) and spectral energy measures, capture only limited acoustic information and are often sensitive to noise. Recent advances in DL, particularly models like ECAPA-TDNN, offer a stronger representation capability by leveraging channel attention and feature aggregation to learn discriminative embeddings from complex, non-stationary signals [7]. Combining Emphasized Channel Attention, Propagation and Aggregation Time Delay Neural Network (ECAPA) with appropriate preprocessing techniques enables more accurate and robust classification of respiratory sounds, providing a promising approach for automated COPD detection [8]. This study does not propose a novel ECAPA-TDNN architecture. Instead, its novelty lies in a hybrid framework that integrates feature selection, robustness-oriented preprocessing, and clinical interpretability, which are not commonly addressed in existing ECAPA-based studies.

The objectives of this work are as follows:

1. To develop a novel adaptation of the ECAPA-TDNN architecture for respiratory sound classification to support automated COPD diagnosis.
2. To design and implement a comprehensive respiratory sound analysis pipeline that integrates advanced preprocessing techniques to enhance signal quality and ensure robust model performance under diverse recording conditions.
3. To apply and optimize feature selection strategies that address class imbalance and improve model generalizability and predictive accuracy across heterogeneous datasets.
4. To enhance the interpretability and clinical applicability of the proposed model through explainable AI techniques, including Grad-CAM visualization.

### 1.2. Related Works

Previous studies have shown that respiratory sound signals can be used to identify pulmonary conditions such as asthma, pneumonia, and COPD. Traditional approaches rely on handcrafted acoustic features like MFCCs and the zero-crossing rate, combined with ML classifiers such as Artificial Neural Network (ANN), SVM, and K-Nearest Neighbor (KNN). Granero mentioned in his work that Decision Tree (DT) can reach 87.8% accuracy in 2018, while Amaral built automated detection workflows using several ML classifiers including SVM and ANN, where SVM achieved the best performance of 96.60% with selected features [9]. However, these approaches are highly dependent on manually engineered features, which may not fully capture the complex temporal and spectral characteristics of respiratory sounds. The strong reliance on handcrafted acoustic features may constrain the models’ capacity to learn complex and subtle acoustic patterns inherent in respiratory sounds, potentially limiting their adaptability across diverse patient populations, recording devices, and clinical environments. In addition, their performance may degrade when applied to more diverse datasets or real-world clinical environments due to limited generalizability. Therefore, while traditional ML-based methods demonstrate promising results, their reliance on handcrafted features and limited robustness highlights the need for more advanced DL approaches.

Specifically, DL-based approaches, particularly CNN and RNN, have been widely adopted to learn representations directly from spectrograms. CNN-based models are effective in capturing local time–frequency patterns, whereas RNN-based models are capable of modeling temporal dependencies. However, CNNs often struggle with long-range temporal relationships, and RNNs can suffer from high computational cost and training instability.

Despite these advances, existing methods still face challenges in effectively capturing multi-scale temporal dynamics and maintaining robustness under limited and imbalanced datasets. To address these limitations, this study adopts the ECAPA-TDNN architecture, which integrates channel attention, multi-scale feature aggregation, and attentive pooling, providing a more efficient and robust solution for respiratory sound classification [9].

With the emergence of DL, CNN and recurrent models have been increasingly applied to spectrogram representations of lung sounds [10]. Zhang employed an innovative model with existing Vit model and can improve the whole metric performance with 99.3% accuracy. However, most of these models struggle to capture complex hierarchical and temporal dependencies.

The ECAPA-TDNN model, originally developed for speaker recognition, integrates channel attention and residual blocks with attentive statistical pooling [11]. However, most existing ECAPA-TDNN studies focus primarily on model architecture, while feature-level optimization, robustness evaluation, and interpretability remain underexplored. It has shown success in various audio tasks, but its use in medical respiratory sound classification remains limited. This study explores its application for automatic COPD detection using the Kaggle Respiratory Sound Database.

In addition to respiratory sound-based approaches, imaging modalities such as chest X-ray (CXR) and low-dose computed tomography (LDCT) have also been widely explored for COPD detection. These methods, including radiomic and deep learning-based techniques, can effectively capture structural lung abnormalities such as emphysema and airway remodeling [12].

However, such imaging-based approaches typically require specialized equipment, higher costs, and may involve exposure to ionizing radiation, which can limit their applicability for large-scale screening and continuous monitoring. In contrast, respiratory sound analysis provides a non-invasive, low-cost, and easily accessible alternative, making it particularly suitable for early screening and deployment in resource-limited settings [13].

Therefore, while imaging-based methods remain important in clinical diagnosis, this study focuses on respiratory sound analysis due to its practicality and scalability [14]. Respiratory sound analysis offers a non-invasive, cost-effective, and easily deployable approach for continuous monitoring and early detection of pulmonary conditions, making it particularly suitable for large-scale screening and resource-limited settings.

### 1.3. Contributions

The main contributions of this work are summarized as follows:

- A hybrid framework is proposed by integrating LassoCV-based feature selection with ECAPA-TDNN, enabling more effective learning from high-dimensional respiratory spectrograms.
- A robust preprocessing pipeline is designed, including segmentation, augmentation, and patient-wise data splitting, to improve generalization under limited and imbalanced data conditions.
- The model’s robustness is systematically evaluated under multiple noise levels, demonstrating stable performance in realistic acoustic environments.
- Grad-CAM-based interpretability is incorporated to provide clinically meaningful visualization of discriminative respiratory patterns.

## 2. Materials and Methods

**Figure 1:**
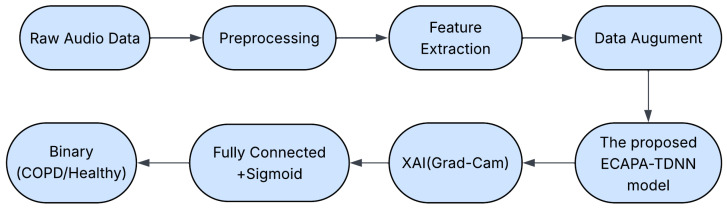
Pipeline of the proposed COPD detection system based on respiratory sounds.

Figure 1 and Table 1 present the complete processing pipeline used for COPD classification from respiratory sounds. The system starts with raw WAV recordings, which undergo preprocessing steps including resampling and time segmentation. Log-Mel spectrograms are computed to transform the waveform into a perceptually meaningful frequency domain. Data augmentation methods such as SpecAugment and noise injection are applied to increase variability and prevent overfitting. The pipeline is divided into four main stages: preprocessing, feature extraction, model training, and evaluation. The processed spectrograms are then input into the ECAPA-TDNN architecture, where SE-Res2Blocks and attentive pooling extract high-level respiratory sound embeddings. These embeddings are passed through a fully connected layer with a sigmoid function to produce the binary output (Healthy or COPD). To provide model interpretability, Grad-CAM is applied to the ECAPA feature maps, highlighting spectral regions that are most influential for the classification decision.

### 2.1. Dataset

We employed the International Conference on Biomedical and Health Informatics (ICBHI) respiratory sound database, publicly available via Kaggle (https://www.kaggle.com/datasets/vbookshelf/respiratory-sound-database, accessed on 24 May 2025), which consists of 920 audio recordings collected from 126 patients across multiple respiratory conditions [15]. This diverse and well-annotated dataset enables the development and evaluation of robust respiratory sound analysis models capable of distinguishing between a variety of pulmonary abnormalities. Each recording is accompanied by annotations in a corresponding text file. For this study [16], we focused exclusively on a binary classification task involving COPD and healthy controls. Diagnosis labels were retrieved from the official metadata file [15]. The final subset used in our experiments includes 64 patients with COPD and 26 healthy individuals.

The dataset contains information on 125 patients, each identified by a unique patient ID and a corresponding medical diagnosis. The dataset includes multiple diagnostic categories such as healthy, asthma, and COPD. This paper focused on the 26 Healthy patients and 64 COPD patients, totaling 793 audio files from COPD patients and 35 audio files from healthy individuals. Creating a binary classification subset is useful for medical or machine learning studies. This allows for comparative analysis or predictive modeling to distinguish between healthy individuals and those suffering from COPD.

**Figure 2:**
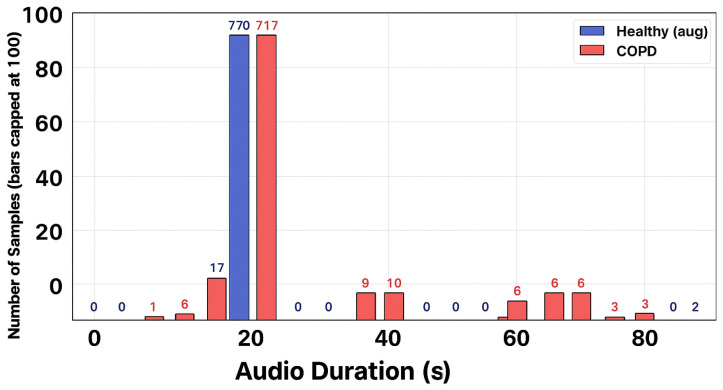
Audio duration distribution (healthy vs. COPD).

The dataset in Figure 2 consists of respiratory sound recordings from healthy and COPD subjects, with most samples concentrated around 20 s in duration and a smaller number of longer COPD recordings.

**Figure 3:**
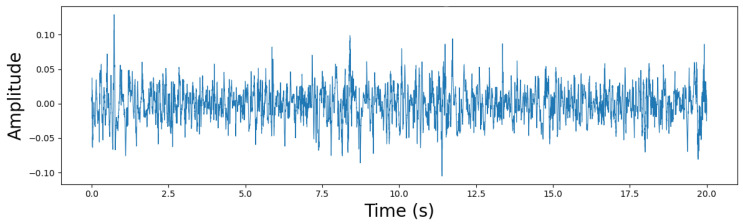
Waveform of healthy patients.

**Figure 4:**
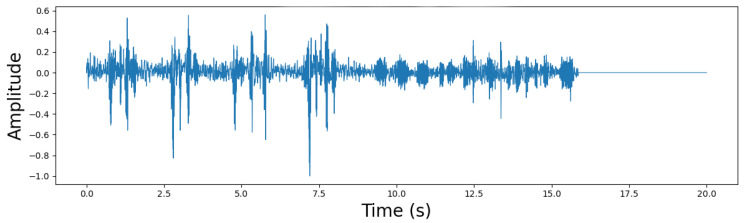
Waveform of COPD patients.

Recordings were downsampled to 16 kHz and segmented into nonoverlapping 5-s clips. A patient-wise split was then performed to create subject-independent training, validation, and test sets in Figure 3 and Figure 4. To mitigate class imbalance, oversampling was applied only to the training set by repeating minority-class samples, while the validation and test sets remained unchanged. After augmentation, the audio duration distribution showed that most recordings for both groups were around 20 s long, while COPD samples exhibited greater variability with some longer recordings. This process helped balance the dataset for subsequent analysis and model training while maintaining the natural diversity of the COPD data.

This approach reflects clinical practice, where diagnosis is based on multiple respiratory observations rather than a single short segment.

Respiratory recordings were segmented into non-overlapping 5-s clips. This duration was chosen as a compromise between capturing sufficient temporal context for clinically relevant events (e.g., wheezes and crackles) and increasing the number of training samples. Shorter segments may miss complete acoustic patterns, while longer segments may introduce redundant or silent regions.

### 2.2. Data Splitting Strategy

**Figure 5:**
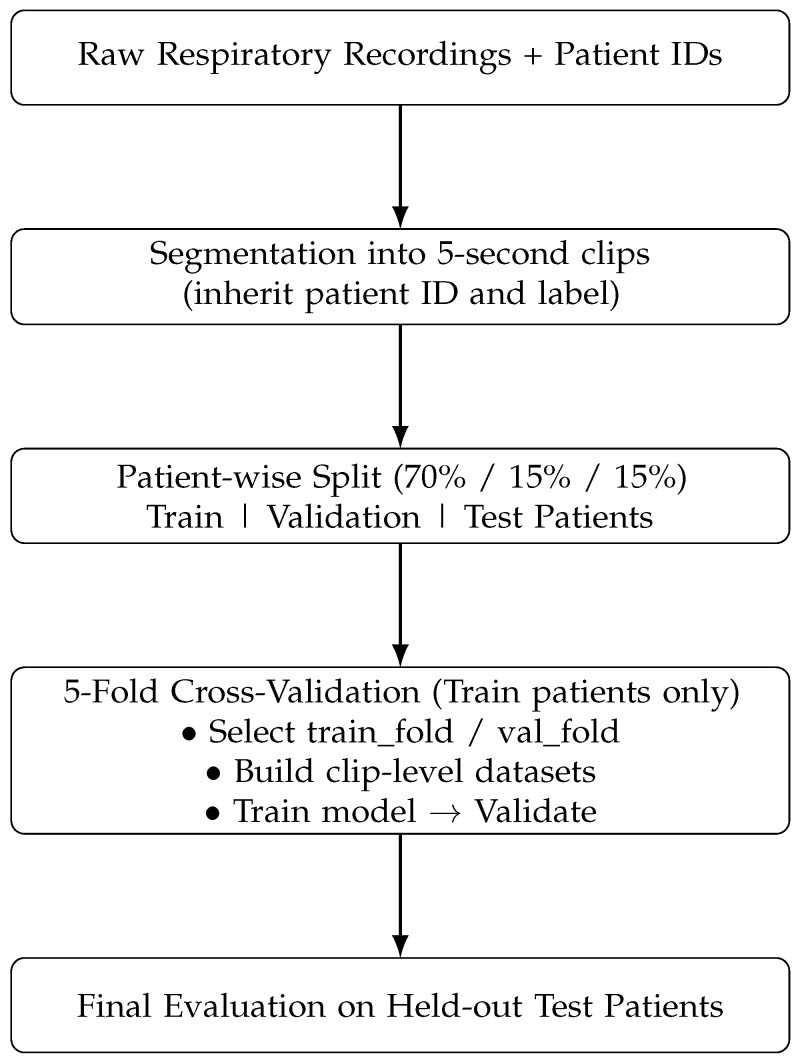
Block diagram of the patient-wise data splitting and cross-validation framework.

The dataset in Figure 5 was partitioned at the patient level into training, validation, and test sets, with an approximate ratio of 70%, 15%, and 15%, respectively. This splitting strategy ensures that samples from the same patient do not appear in multiple subsets, thereby preventing data leakage and providing a reliable evaluation of model generalization. Recordings were segmented into non-overlapping 5-s clips to standardize input length. All segments derived from the same patient were kept within the same data split (training, validation, or test) to prevent data leakage and ensure subject-independent evaluation.

In addition, cross-validation was employed to monitor model stability and mitigate overfitting. The consistent performance observed across folds further suggests that the impact of oversampling on generalization is limited [17]. It provides a systematic framework to evaluate model robustness across diverse subsets of the dataset, ensuring consistent performance under varying real-world conditions.

To mitigate this limitation, we strengthened the experimental setup in the revised manuscript. Specifically, the number of training epochs was increased from 5 to 50 to ensure more stable convergence, and early stopping (patience = 3) was applied to prevent overfitting. In addition, patient-wise splitting and 5-fold cross-validation were employed to provide a robust evaluation and reduce potential bias.

### 2.3. Feature Extracture

**Figure 6:**
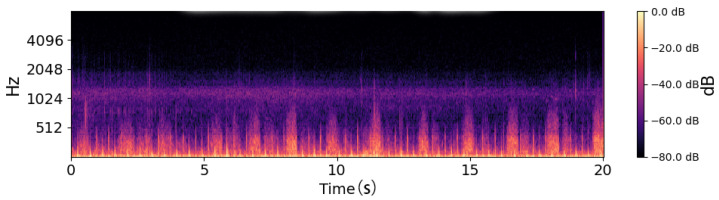
Log-Mel spectrogram of a healthy subject. The spectrogram shows smooth and stable low-frequency energy patterns, corresponding to regular breathing cycles.

The bright regions of Figure 6 in the log-Mel spectrograms represent time–frequency segments with higher acoustic energy, shown in yellow or bright orange. These areas correspond to moments where airflow intensity increases during the breathing cycle, such as during deeper inhalation or exhalation [18], or when turbulence occurs in the respiratory tract. In respiratory sound analysis, these high-energy components are important because they reflect how air moves through the airways and how much resistance or turbulence is present [19].

**Figure 7:**
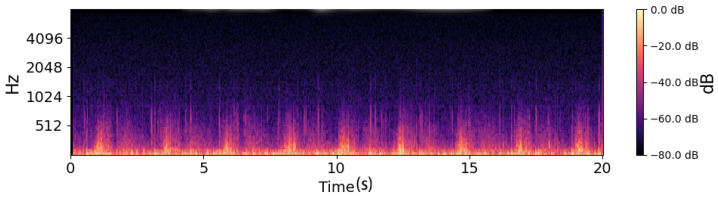
Log-Mel spectrogram of a COPD patient. The spectrogram exhibits irregular energy distributions with intermittent high-frequency components, reflecting abnormal respiratory sounds such as wheezes and crackles.

Compared to the healthy subject, the COPD patient in Figure 7 exhibits noticeably fewer and more irregular bright regions, which are mostly confined to lower frequencies. This pattern reflects impaired airflow consistency and attenuation of mid-to-high-frequency components, indicative of airway obstruction. In contrast, the healthy subject shows brighter, more continuous energy bands across a wider frequency range, reflecting stable breathing and unobstructed airflow. These differences in the distribution and intensity of bright regions provide discriminative acoustic features that support binary classification between healthy and COPD respiratory sounds.

### 2.4. Model Architecture

**Figure 8:**
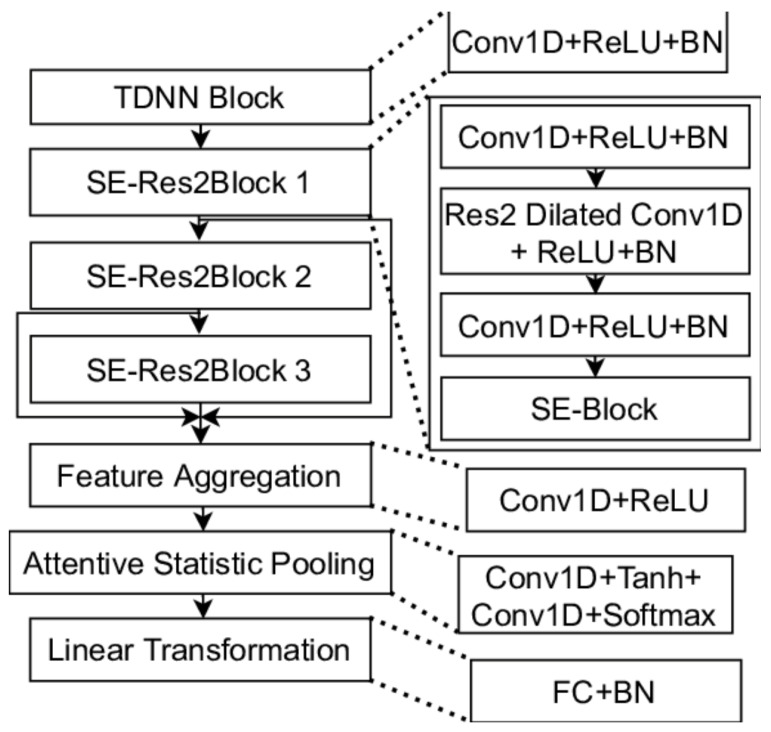
Architecture of the proposed ECAPA-TDNN model.

In this essay, the main model is ECAPA-TDNN in Figure 8, which is a compact 1-D conv architecture well-suited to cough-based respiratory screening: a TDNN front-end feeds stacked SE-Res2Blocks (Res2Net multi-scale convolutions with residual paths and SE channel attention) and comparison among those models is in Table 2, whose outputs are fused by multi-layer feature aggregation and compressed by Attentive Statistics Pooling (ASP) into an attention-weighted mean/variance vector; a linear projection yields stable embedding for a lightweight classifier [20]. This design captures both the burst–decay micro-dynamics of coughs and slower envelopes, while SE emphasizes informative spectral patterns. ASP focuses the representation on key frames, improving discriminability over plain averaging. Starting from a pretrained ECAPA encoder enables strong performance with limited labeled cough data and allows for optional fine-tuning of later blocks. The resulting embeddings aggregate cleanly across multiple coughs per subject [21], supporting calibrated patient-level decisions with fast inference.

In addition, LassoCV-based feature selection is incorporated as a complementary step for feature analysis within the pipeline, without replacing the end-to-end representation learning of the ECAPA-TDNN model.

Multi-scale temporal convolutions (Res2Net) capture the burst–decay micro-dynamics of coughs. The Time Delay Neural Network (TDNN) context models short- to mid-range temporal patterns. ASP highlights key frames (e.g., burst/closure), preserving discriminative cues. And squeeze-and-excitation (SE) channel attention reweights spectral channels to emphasize informative frequency patterns [22].

Using a pretrained ECAPA encoder + lightweight classification head then optionally fine-tuning later blocks is effective even with limited labeled cough data. It produces stable clip embeddings that aggregate well across multiple coughs per subject (mean/attention). It plays well with class-imbalance strategies (Focal Loss, class weights) and patient-wise splits to avoid leakage.

Compact, straightforward 1-D conv + ASP architecture with fast inference.

Easy calibration (temperature scaling + threshold tuning) to hit sensitivity/specificity targets.

Built-in attention weights/heat maps support interpretability for review and audit.

Combining SE channel attention with Res2Net structure enhances modeling of inter-channel dependencies in feature maps [23].

In ECAPA-TDNN for cough screening, 1D convolutions aggregate local temporal context from raw waveforms to capture the cough’s burst–decay transients and envelope patterns. The TDNN/front-end and Res2 (with dilated convs) provide multi-scale temporal modeling, covering short transients and longer contexts. The 1 × 1 convolutions perform channel mixing and dimensionality reduction, recombining features into more discriminative representations. In ASP, a small conv network produces frame-wise attention weights to compress variable-length sequences into an attention-weighted global vector. Overall, conv layers jointly handle temporal modeling, channel reweighting, and attention scoring to yield stable, classifiable embeddings with parameter efficiency and low latency [24]. The input to the model is a log-Mel spectrogram with 80 frequency bins, as summarized in Table 3. An optional frequency gating mechanism is applied to adaptively reweight each frequency band. The ECAPA-TDNN model was implemented with a channel size of 1024 and an embedding dimension of 192. The input to the model consists of log-Mel spectrograms with 80 frequency bins, capturing the time–frequency characteristics of respiratory sounds. Each convolutional layer is followed by batch normalization and ReLU activation, and squeeze-and-excitation (SE) blocks are incorporated to enhance channel-wise feature representation. Attentive Statistics Pooling (ASP) is employed to aggregate frame-level features into a fixed-length representation [23]. Residual connections within the ECAPA-TDNN architecture further facilitate gradient flow, improving training stability and the extraction of discriminative features from respiratory sounds. The final fully connected layer outputs two classes for binary classification. These settings provide sufficient model capacity to capture complex acoustic patterns while maintaining stable training.

### 2.5. Attentive Statistics Pooling

ASP is employed to aggregate frame-level representations into a fixed-dimensional embedding. Unlike conventional statistical pooling methods that compute simple mean and standard deviation, ASP introduces an attention mechanism to assign different importance weights to each temporal frame.

Specifically, given a sequence of frame-level features, an attention layer is applied to generate normalized weights, highlighting informative segments while suppressing less relevant or noisy regions [25]. The weighted mean and weighted standard deviation are then computed as

(1) $$\mu =\sum_{t}\alpha_{t}h_{t},\;\sigma =\sqrt{\sum_{t}\alpha_{t}{\left(h_{t}-\mu \right)}^{2}}$$

where $h_{t}$ denotes the feature at time step *t*, and $\alpha_{t}$ represents the attention weight satisfying $\sum_{t}\alpha_{t}=1$. By focusing on acoustically salient regions, ASP enhances the model’s ability to capture discriminative temporal patterns, which is particularly beneficial for respiratory sound analysis, where abnormal events (e.g., wheezes or crackles) occur intermittently.

### 2.6. LassoCV

It is important to clarify the interaction between LassoCV-based feature selection and the ECAPA-TDNN model. In this study, LassoCV was applied to the flattened log-Mel spectrogram features prior to model training in order to identify the most discriminative time–frequency components. Specifically, the 80-bin log-Mel spectrogram was reshaped into a high-dimensional feature vector for each sample. LassoCV was then used to eliminate redundant or low-contribution spectral bins by shrinking insignificant coefficients to zero [26]. The retained features were subsequently reshaped back to the original time–frequency structure and used as the input to the ECAPA-TDNN network [27]. Therefore, LassoCV serves as a pre-selection mechanism operating at the spectrogram level, while ECAPA-TDNN performs hierarchical temporal modeling on the filtered representation. This design ensures that ECAPA focuses on acoustically informative regions, potentially improving generalization under high-dimensional and imbalanced conditions.

To reduce dimensionality and improve interpretability, we employed the Least Absolute Shrinkage and Selection Operator (LASSO) with cross-validation (LassoCV) as a sparse feature selection mechanism.

Let the standardized flattened log-Mel spectrogram features be denoted as $X\in R^{n\times p}$ and the label vector as $y\in R^{n}$, where *n* represents the number of samples and *p* denotes the number of extracted features. The linear regression model can be written as [28]

(2) y=Xβ+ε,

where $\beta \in R^{p}$ is the coefficient vector and ε is the error term.

Unlike ordinary least squares (OLS), LASSO introduces an $L_{1}$ regularization term to promote sparsity in the solution. The optimization objective is defined as [29]

(3) $$\hat{\beta }=\arg\min_{\beta }\left(\frac{1}{2n}{∥y-X\beta ∥}_{2}^{2}+\lambda {∥\beta ∥}_{1}\right),$$

where ${∥\beta ∥}_{1}=\sum_{j=1}^{p}\left|\beta_{j}\right|$ and λ≥0 is the regularization parameter controlling the trade-off between data fidelity and sparsity.

The $L_{1}$ penalty induces sparsity by shrinking small coefficients toward zero. In the orthogonal design case, the solution reduces to a soft-thresholding operator [30]:

(4) $${\hat{\beta }}_{j}=\mathrm{sign}\left(\beta_{j}^{OLS}\right)\max\left(|\beta_{j}^{OLS}|-\lambda ,0\right),$$

Only features with non-zero coefficients were retained for subsequent training of the ECAPA-TDNN classifier. This approach reduces redundant or noisy time–frequency components in the high-dimensional spectrogram space, mitigates overfitting, and improves computational efficiency. To reduce dimensionality and enhance the model’s interpretability, we employed Lasso regression with cross-validation (LassoCV) as a feature selection technique [31].

Repetition-based oversampling may increase the risk of overfitting due to duplicate samples. However, in this study, oversampling was applied exclusively to the training data within each cross-validation fold, while validation and test sets remained unchanged. This design ensures that performance evaluation reflects true generalization.

### 2.7. Noise Robustness

To simulate real-world acoustic conditions, Gaussian noise was introduced to the input mel spectrograms at different signal-to-noise ratio (SNR) levels. By controlling the SNR, varying degrees of background noise were artificially injected into the respiratory sound representations, ranging from severe noise interference to near-clean conditions. By incorporating varying noise levels, the model becomes capable of handling diverse acoustic scenarios without significant degradation in accuracy. This process allows the model to be evaluated under progressively more challenging environments, reflecting practical scenarios such as clinical settings or home monitoring where recordings are often affected by ambient noise. The incorporation of noise at different levels provides a controlled framework to examine how the model responds to signal degradation and to assess its stability and reliability when exposed to noisy inputs.

To simulate realistic noisy environments, Gaussian noise was injected into the input mel-spectrograms based on a predefined signal-to-noise ratio (SNR) [32]. The SNR in decibels is defined as

(5) $${\mathrm{SNR}}_{\mathrm{dB}}=10\log_{10}\left(\frac{P_{\mathrm{signal}}}{P_{\mathrm{noise}}}\right)$$

where $P_{\mathrm{signal}}$ and $P_{\mathrm{noise}}$ represent the power of the original signal and the injected noise, respectively. Given a target SNR level, the corresponding noise power is computed as [32]

(6) $$P_{\mathrm{noise}}=\frac{P_{\mathrm{signal}}}{10^{{\mathrm{SNR}}_{\mathrm{dB}}/10}}$$

Subsequently, Gaussian noise $n∼N(0,P_{\mathrm{noise}})$ is generated and added to the input signal *x*, resulting in a noisy observation $\tilde{x}$ [33]:

(7) $$\tilde{x}=x+n$$

By varying the SNR levels, different degrees of noise contamination were introduced, ranging from highly degraded to near-clean conditions. This formulation enables controlled perturbation of the input data, allowing the evaluation of model behavior under signal degradation and providing insights into its robustness and stability in practical respiratory sound analysis scenarios.

### 2.8. GRADCAM

We apply Grad-CAM on ECAPA-TDNN (layer4, before ASP) to highlight time regions that contribute most to the target score, overlaid on the 80-bin log-Mel spectrogram. COPD recordings exhibit recurrent activations concentrated within specific frequency ranges across multiple respiratory cycles [34].

**Figure 9:**
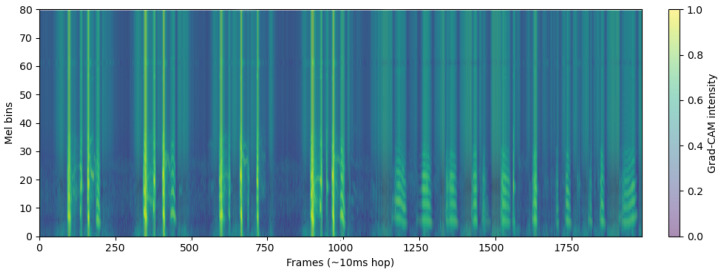
ECAPA-TDNN layer4 Grad-CAM of COPD patient.

The Grad-CAM maps reveal clear class-dependent patterns.COPD recordings in Figure 9 show persistent, band-limited hotspots that recur across breathing cycles, mainly in the low–mid bands with occasional upward spread—wheeze-like evidence indicating stable abnormal airflow. Hotspots appear intermittently and occupy slimmer frequency bands, consistent with intermittent wheeze.

**Figure 10:**
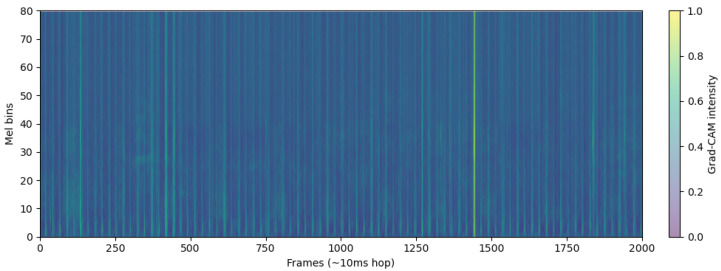
ECAPA-TDNN layer4 Grad-CAM of healthy patient.

The healthy recordings shown in Figure 10 display overall low, scattered intensity with small peaks around normal inhale/exhale transitions and no long, coherent bands, suggesting an absence of pathological cues.

Grad-CAM intensity ranks COPD > Healthy in coverage, temporal continuity, and band prominence. This alignment with expected respiratory acoustics indicates that the ECAPA-TDNN relies on clinically meaningful cues rather than noise and provides time-localized evidence that aids reviewer verification. For a compact quantitative check, we report the hot-zone ratio (percentage of frames with CAM≥0.6) and the mean contiguous run length of hot segments; both should follow the same ordering [35,36].

### 2.9. Training Configuration

Table 4 summarizes the key training hyperparameters and experimental settings used in this study to ensure reproducibility. The model was trained using the AdamW optimizer with a fixed learning rate and batch size over a predefined number of epochs. Early stopping was applied based on validation performance to prevent overfitting, and the final model was selected according to the best validation F1-score. All experiments were conducted on a CUDA-enabled GPU [37].

### 2.10. Evaluation

To evaluate the proposed model, we employed 5-fold cross-validation, ensuring that each data subset served as the validation set exactly once while the others were used for training. The model’s performance was assessed at each epoch using the following standard classification metrics: accuracy, precision, recall, F1-score, and loss. These metrics were calculated separately on both the training set and the validation set for each fold and epoch.

Performance was tracked throughout training to monitor learning dynamics and potential overfitting. The final evaluation was based on the average validation metrics across folds. This approach provides a robust estimate of generalization performance and mitigates bias from any single train–test split.

## 3. Results

**Figure 11:**
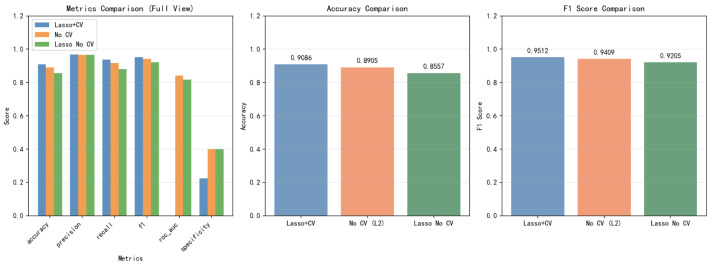
Comparison of model performance under different feature selection and validation strategies, showing that Lasso+CV provides the best overall results.

All reported performance metrics are obtained from the independent test set. Cross-validation was used only for model development, including hyperparameter tuning. Figure 11 presents the performance comparison of three modeling strategies, including Lasso with cross-validation (Lasso+CV), no cross-validation (No CV, L2), and Lasso without cross-validation.

Specifically, Lasso+CV attains the highest accuracy of 90.86%, compared to 89.05% for No CV and 85.57% for Lasso without CV. A similar trend is observed in the the F1-score, where Lasso+CV achieves 95.12%, outperforming No CV 94.09% and Lasso without CV 92.05%.

In addition, Lasso+CV demonstrates improved recall and ROC-AUC, indicating enhanced sensitivity and discriminative capability. In contrast, models without cross-validation or without Lasso-based feature selection exhibit relatively lower and less consistent performance.

**Figure 12:**
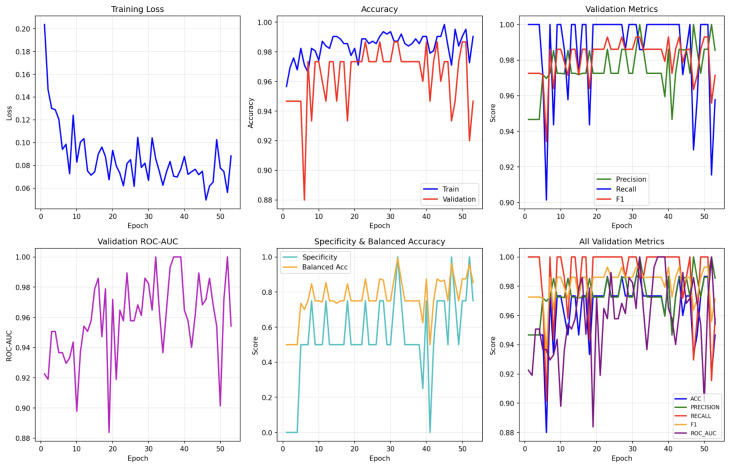
Training dynamics across 50 epochs, showing the evolution of loss, accuracy, ROC-AUC, and validation metrics. The curves indicate stable optimization and effective convergence without significant overfitting.

The training dynamics of the proposed model over 50 epochs are illustrated in Figure 12. The training loss shows a rapid decrease during the initial epochs followed by a gradual stabilization, indicating effective optimization. Simultaneously, the validation loss remains relatively steady, suggesting that the model avoids overfitting and maintains generalization capability throughout the training process. Both training and validation accuracies remain consistently high, with only minor fluctuations, suggesting good generalization performance.

The validation metrics, including precision, recall, F1-score, and ROC-AUC, exhibit stable trends across epochs, further confirming the robustness of the model. Importantly, no significant divergence between training and validation curves is observed, implying that overfitting is effectively mitigated through early stopping and regularization strategies. Overall, the curves demonstrate that the model converges reliably and maintains consistent performance throughout training.

**Figure 13:**
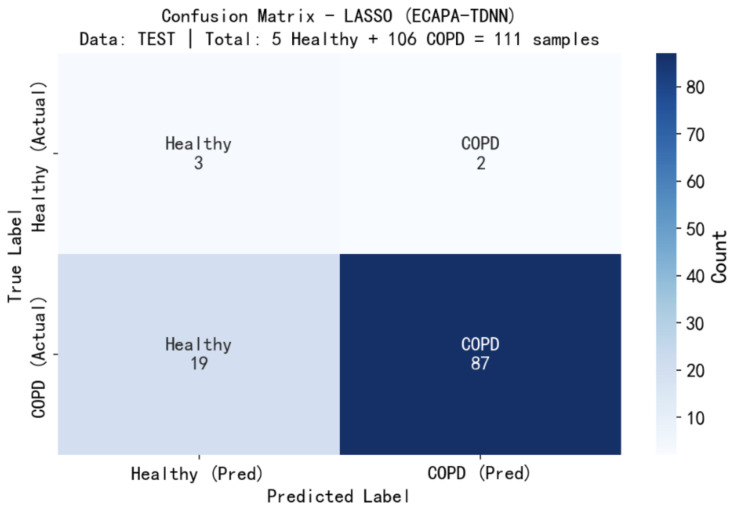
Confusion matrix.

Figure 13 presents the confusion matrix of the proposed LASSO + ECAPA-TDNN model evaluated on the independent test set. The test set consists of 111 samples, including 5 healthy subjects and 106 COPD patients.

In accordance with Figure 13, the confusion matrix highlights the model’s classification performance, showing high true positive rates for COPD cases while maintaining accurate identification of healthy subjects, thereby demonstrating its effectiveness in distinguishing between the two classes. It shows that three healthy samples were correctly classified, while two were misclassified as COPD. For the COPD class, 87 samples were correctly identified and 19 were incorrectly predicted as healthy. These results indicate a higher sensitivity for detecting COPD cases compared to healthy subjects, highlighting a slight class imbalance effect on model performance and suggesting areas for potential improvement in distinguishing mild or borderline cases.

The overall classification accuracy achieved on the test set is 81.1%. The sensitivity (recall) for COPD is 82.1%, indicating that the model demonstrates strong detection capability for COPD patients. The specificity for the healthy class is 60.0%, suggesting relatively lower performance in distinguishing healthy individuals.

It is noteworthy that the dataset is highly imbalanced, with significantly fewer healthy samples compared to COPD samples. This class imbalance may contribute to the reduced specificity observed for the minority class. Overall, the proposed model shows satisfactory performance in detecting COPD, while further improvements are needed to enhance recognition of the minority class.

### 3.1. Ablation Study

To investigate the contribution of each module in the proposed framework, an ablation study was performed, and the results are summarized in Table 5. The full model achieved an accuracy of 90.86%, an F1-score of 95.1%, and a recall of 94.0%, indicating strong overall classification performance. The ablation results further confirm that the integration of all modules synergistically enhances feature representation and classification capability, highlighting the importance of the complete model architecture for robust respiratory sound analysis.

When Attentive Statistics Pooling (ASP) was removed, the F1-score decreased to 91.8% and recall dropped to 90.7%, demonstrating that attentive temporal aggregation plays a crucial role in capturing discriminative respiratory sound patterns. This decline in performance underscores the importance of ASP in effectively summarizing frame-level features into a fixed-length representation, enabling the model to accurately differentiate between subtle variations in respiratory sounds.

Removing the LassoCV feature selection module resulted in a decline in the F1-score to 93.2% and recall to 92.1%, suggesting that sparse feature selection enhances generalization by eliminating redundant and noisy features. The performance drop highlights the role of LassoCV in identifying the most informative features, thereby improving model efficiency and preventing overfitting to irrelevant or noisy data.

Similarly, excluding the SE module led to performance degradation (F1: 92.3%, Recall: 89.7%), highlighting the importance of channel-wise attention in emphasizing informative frequency bands relevant to COPD detection. The observed decrease in performance indicates that without the SE module, the model struggles to effectively prioritize critical frequency information, reducing its overall classification accuracy.

Overall, the comparison in Table 5 confirms that each component contributes positively to the final performance, and their integration enables the proposed model to achieve optimal classification effectiveness. These results emphasize that the combined effect of all modules is greater than the sum of individual contributions, demonstrating the necessity of the full architecture for robust and accurate respiratory sound classification.

Table 6 presents the performance comparison of different modeling strategies. Overall, the Lasso+CV approach consistently achieves the best results across all evaluation metrics.

Specifically, Lasso+CV attains the highest accuracy of 90.86%, outperforming No CV (89.05%) and Lasso without CV (85.57%). A similar trend is observed in the F1-score, where Lasso+CV achieves 95.12%, compared to 94.09% for No CV and 92.05% for Lasso without CV.

In terms of recall, Lasso+CV also demonstrates superior sensitivity (93.65%), indicating improved capability in detecting positive cases. Precision remains consistently high across all methods (approximately 96%), suggesting stable performance in avoiding false positives.

Overall, these results indicate that combining Lasso-based feature selection with cross-validation enhances model robustness and generalization performance.

### 3.2. Noisy Robustness

Table 7 presents the noise robustness evaluation of the proposed model under varying SNR conditions. The model maintains stable performance at higher SNR levels (15–30 dB), achieving an accuracy of approximately 90%, which indicates strong robustness to moderate noise.

As the noise level increases, a gradual decline in performance is observed. Specifically, the accuracy decreases to 83.33% at 10 dB and further to 80.00% at 0 dB. A slight fluctuation is observed at 5 dB, which can be attributed to variability in the dataset and stochastic effects during training [26].

Overall, the results demonstrate that the proposed model remains resilient under noisy conditions and is capable of maintaining reliable performance even in low-SNR environments. This highlights its potential applicability in real-world respiratory sound analysis, where background noise is often unavoidable.

### 3.3. Imbalance Handling Method

To evaluate the impact of class imbalance handling strategies, we compared the performance of the class weighting and oversampling approaches across multiple metrics (Table 8). All compared studies are based on the same respiratory sound dataset to ensure a fair comparison [38].

Oversampling achieved slightly higher overall performance, with an accuracy of 90.86% compared to 87.00% using class weighting [39]. Improvements were also observed in precision (72.86% vs. 72.46%), recall (68.92% vs. 67.57%), and F1 score (70.83% vs. 69.93%). However, the ROC AUC values were comparable between the two methods (52.34% vs. 53.22%), and both approaches yielded identical specificity (26.92%). These results indicate that oversampling provides a modest but consistent improvement in sensitivity-related metrics. This suggests that while oversampling can enhance the detection of minority class instances, careful consideration is still needed to balance overall model performance and avoid potential overfitting.

Repetition-based oversampling does not introduce genuinely new information, as it duplicates existing samples, and may increase the risk of overfitting. However, this risk is mitigated through the use of multiple regularization and training strategies, including dropout, L1 regularization, early stopping, class-weighted loss, and data augmentation.

### 3.4. Grad-CAM

As shown in Table 9, the Grad-CAM maximum activation values ranged from approximately 0.0047 to 0.0059, while the mean activation values were around 0.001. COPD samples demonstrated relatively higher and more stable activation intensities compared with healthy samples. This quantitative analysis supports the reliability of the attention mechanism in highlighting clinically meaningful acoustic features.

Grad-CAM was implemented by computing the gradients of the target class score with respect to the feature maps extracted from the last convolutional layer (layer 4) of the ECAPA-TDNN model. These gradients were globally averaged to obtain channel-wise weights, which were then combined with the feature maps to generate class activation maps. The resulting heat maps were upsampled and overlaid on the log-Mel spectrograms to highlight the most informative time–frequency regions.

## 4. Discussion

Table 10 compares representative COPD detection studies based on respiratory sound analysis. “N/A” means no cross validation has been mentioned in the study. Early work such as Amaral et al. (2012) [40] and Granero et al. (2018) [9] relied on classical ML models with handcrafted features, achieving moderate performance on small private datasets. With the advancement of deep learning, Srivastava et al. (2021) [41] and Alghamdi et al. (2024) [3] employed CNN-based architectures and reported improved performance, and Zhang et al. (2025) [42] built an innovative model with existing technology, called the Vit S model, and its accuracy could reach 99.3%., although limitations in model expressiveness and dataset diversity persisted.More recently, Sharma et al. (2023) [43] applied a CNN to the ICBHI dataset, achieving a sensitivity of 96.59% and specificity of 96.31%.

The reported accuracy of 90.86% corresponds to the test set performance. Although the cross-validation results are higher, the independent test set provides a stricter evaluation. The observed performance gap may be attributed to the limited sample size and distributional differences, which may indicate a degree of model instability. The learning curves over 50 epochs indicate effective convergence without substantial overfitting. Training loss decreases rapidly at early epochs and then stabilizes, while training and validation accuracies remain consistently high with only minor fluctuations. Importantly, the absence of a clear divergence between the training and validation curves suggests that the adopted regularization and early-stopping strategy successfully constrains overfitting. Such stable optimization behavior supports the feasibility of applying the method to limited medical audio datasets, where overfitting is a common risk.

To further understand model behavior, we analyze the false positives and misclassification patterns based on the confusion matrix. The model shows a higher sensitivity toward COPD detection, correctly identifying most COPD cases, while a portion of healthy samples are misclassified as COPD (false positives). This behavior can be attributed to the class imbalance, where COPD samples dominate the dataset, leading the model to favor the majority class. In addition, some healthy recordings may contain mild or ambiguous acoustic patterns that resemble early-stage or subtle respiratory abnormalities, contributing to misclassification. Conversely, a small number of COPD cases are misclassified as healthy (false negatives), which may be due to weak or less pronounced pathological acoustic features in certain recordings. Overall, the misclassification pattern suggests that the model prioritizes detecting COPD (higher recall) at the expense of specificity, which is consistent with screening-oriented applications.

As described in the Methodology Section, LassoCV was used as a sparse spectrogram-level pre-selection mechanism.

Beyond quantitative performance, interpretability plays a critical role in the clinical translation of AI-based diagnostic systems. The Grad-CAM visualizations reveal clear class-dependent activation patterns [44]. COPD recordings exhibit temporally continuous and band-limited hotspots, predominantly in the low-to-mid frequency regions, which are consistent with airflow obstruction and wheeze-like acoustic signatures observed in clinical auscultation. In contrast, healthy recordings demonstrate weaker, scattered activations primarily around normal inhalation–exhalation transitions, suggesting the absence of sustained pathological cues.

The greater temporal continuity and spectral coherence observed in COPD samples indicate that the model relies on stable respiratory abnormalities rather than isolated transient noise artifacts. This observation is further supported by the noise robustness experiments, where performance degradation under low SNR conditions remains gradual rather than abrupt. The preservation of the activation structure across varying noise levels suggests that the model captures intrinsic respiratory acoustic signatures instead of overfitting to high-frequency noise components.

From a decision-boundary perspective, the asymmetry observed in the confusion matrix—higher sensitivity for COPD but lower specificity for healthy—can be partially attributed to dataset imbalance. The predominance of COPD samples likely biases the learned representation toward the majority class, a phenomenon well-documented in imbalanced learning theory. While oversampling mitigates this issue during training, future work may explore threshold calibration or cost-sensitive learning to achieve better class-wise balance, particularly for screening scenarios where minimizing false negatives is clinically prioritized.

Although Grad-CAM provides intuitive visual evidence, it remains a post hoc attribution method and does not establish causal importance. Future studies should incorporate perturbation-based validation, such as frequency masking or occlusion sensitivity tests, to confirm that highlighted spectral–temporal regions are indeed essential for prediction. Such validation would further strengthen clinical trust and support the integration of the proposed framework into physician-in-the-loop diagnostic workflows. Additionally, combining Grad-CAM with quantitative feature importance analyses could provide a more comprehensive understanding of the model’s decision-making process, enhancing interpretability and reliability for clinical applications.

Overall, the integration of multi-scale temporal modeling, channel-wise attention, and sparse feature selection not only improves predictive accuracy but also contributes to physiologically meaningful representation learning, bridging the gap between blue-box deep learning models and interpretable clinical decision support systems.

### 4.1. Limitation

Although the ECAPA-TDNN model demonstrates outstanding performance on the ICBHI dataset, several limitations must be acknowledged.

First, the dataset size is relatively small, and the recordings were collected under controlled conditions using specific recording devices. This limits the model’s ability to generalize to diverse real-world environments, such as varying microphone quality, ambient noise, and patient posture during recording. Future work should explore domain adaptation and noise-robust training strategies to improve generalization [45].

Second, while the current study focuses solely on COPD vs. healthy classification, real-world respiratory diagnostics often involve multiple overlapping conditions, such as asthma and pneumonia. Expanding the model to handle multi-class classification and comorbidities would improve its clinical applicability [46]. Addressing this limitation by including a broader range of respiratory conditions in the training dataset would enable the model to better reflect real-world clinical scenarios and support more comprehensive diagnostic decision-making.

Third, the interpretability of deep models remains limited. Although ECAPA-TDNN includes attention mechanisms, further efforts are required to visualize and explain which acoustic patterns influence the model’s predictions. Integrating Explainable Artificial Intelligenc (XAI) methods, such as Grad-CAM or SHapley Additive exPlanations (SHAP) for spectrograms, could enhance trust among clinicians [47].

### 4.2. Confidence Interval

As shown in Table 11, the proposed model demonstrates strong and stable performance across key evaluation metrics. The accuracy achieves a mean value of 90.86% with a relatively narrow confidence interval, indicating consistent performance across folds. Similarly, precision, recall, and F1-score all exhibit high mean values exceeding 93%, with tight 95% confidence intervals, suggesting the robustness and reliability of the model in identifying COPD cases. Overall, these results confirm the effectiveness of the proposed approach under cross-validation settings.

### 4.3. Future Work

Beyond the experimental setting, integrating AI-based respiratory sound classifiers into real-world clinical workflows presents several challenges. Performance may degrade under uncontrolled acquisition conditions such as heterogeneous microphones, background noise, or patient posture variations, leading to potential domain shift and calibration drift. Deployment further requires clearly defined intended use (screening vs. diagnostic support), appropriate operating thresholds, and quality-control mechanisms to detect low-quality or out-of-distribution inputs. In addition, clinical adoption depends on interpretability, human-in-the-loop verification, and regulatory compliance, including bias assessment and prospective validation in representative clinical environments [48].

Future work will focus on conducting external validation using independent respiratory sound datasets (e.g., Coswara) to evaluate cross-dataset generalization and domain shift effects arising from different recording devices and populations. In addition, prospective validation under real clinical recording conditions will be performed, together with threshold calibration and robustness analysis, to ensure reliable deployment in practical screening scenarios [49].

## 5. Conclusions

This study presents a robust and interpretable deep learning framework for automated COPD detection based on respiratory sound analysis. By combining ECAPA-TDNN with Lasso-based sparse feature selection and Attentive Statistics Pooling, the proposed approach effectively captures discriminative time–frequency patterns associated with airflow obstruction.

Experimental evaluation on the ICBHI dataset demonstrates that the integrated Lasso+CV configuration achieves improved classification performance compared with baseline variants. The ablation study confirms the positive contribution of each architectural component, while noise robustness experiments indicate stable performance under varying acoustic conditions. Importantly, Grad-CAM visualization provides clinically meaningful evidence, showing that the model focuses on physiologically relevant respiratory signatures [50].

Overall, the proposed framework offers a reliable and interpretable solution for AI-assisted respiratory sound classification. These findings highlight the potential of deep learning-based acoustic analysis as a practical and scalable tool for early COPD screening and computer-aided respiratory assessment.

## Figures and Tables

**Table 1:** Summary of the processing pipeline.

Stage	Description
Preprocessing	Audio resampling, segmentation, and normalization
Feature Extraction	Log-Mel spectrogram generation
Model Training	ECAPA-TDNN training with cross-validation
Evaluation	Performance assessment using metrics (accuracy, F1-score, etc.)

**Table 2:** Comparison of TDNN, x-vector, and the proposed ECAPA-TDNN architectures.

Architecture	Key Characteristics
TDNN	Basic time-delay network used to model temporal sequences
x-vector	TDNN-based, adds statistical pooling to extract embeddings
The proposed ECAPA-TDNN model	Adds channel attention, Res2Net blocks, and better aggregation mechanisms

**Table 3:** Architecture configuration of the proposed ECAPA-TDNN model.

Layer	Kernel	In-Ch	Out-Ch	Output Shape
Input (Mel-Spectrogram)	–	–	80	[B,80,T]
Freq Gate (optional)	–	80	80	[B,80,T]
Conv1D + BN + ReLU	5	80	1024	[B,1024,T]
SE Module	1	1024	1024	[B,1024,T]
Conv1D + BN + ReLU	3	1024	1024	[B,1024,T]
SE Module	1	1024	1024	[B,1024,T]
Conv1D + BN + ReLU	3	1024	1024	[B,1024,T]
SE Module	1	1024	1024	[B,1024,T]
Global Avg Pooling	–	1024	1024	[B,1024]
Dropout (p=0.2)	–	1024	1024	[B,1024]
Fully Connected (Embedding)	–	1024	192	[B,192]
Classifier	–	192	2	[B,2]

**Table 4:** Key training hyperparameters for reproducibility.

Parameter	Setting
Optimizer	AdamW
Learning rate	$5\times 10^{-4}$
Batch size	32
Epochs	50
Weight decay	0.0
Early stopping	Patience = 3
Model selection	Best validation F1-score
Training hardware	CUDA-enabled GPU

**Table 5:** Ablation study of the proposed model.

Model	Acc	F1	Recall
Full Model	90.86	95.1	94.0
w/o ASP	93.5	91.8	90.7
w/o LassoCV	91.0	93.2	92.1
w/o SE	89.2	92.3	89.7

**Table 6:** Performance comparison of different methods (%).

Metric	Lasso+CV	No CV	Lasso No CV
Accuracy	90.86%	89.05%	85.57%
Precision	96.71%	96.69%	96.55%
Recall	93.65%	91.62%	87.96%
F1-score	95.12%	94.09%	92.05%

**Table 7:** Model performance under different noise levels.

SNR (dB)	Accuracy (%)
30	90.00
20	90.00
15	90.00
10	83.33
5	86.67
0	80.00

**Table 8:** Comparison with previous studies on the same respiratory sound dataset for COPD classification.

Method	Accuracy (%)	Precision (%)	Recall (%)	F1 Score (%)	ROC AUC (%)	Specificity (%)
Class Weight	87.00	72.46	67.57	69.93	53.22	26.92
Oversampling	90.86	72.86	68.92	70.83	52.34	26.92

**Table 9:** Grad-CAM summary statistics for selected samples.

Sample ID	True Label	Predicted Label	Grad-CAM Max	Grad-CAM Mean
0	1	1	0.0052	0.0010
1	0	0	0.0049	0.0009
2	0	0	0.0059	0.0011
3	1	1	0.0058	0.0011
4	1	1	0.0047	0.0009

**Table 10:** Comparison of the proposed model with existing methods on respiratory sound analysis.

Author, Year	Type	Alg.	Performance	Data	Dataset	CV
Srivastava et al. 2021 [41]	DL	CNN	Acc. = 93%	Pub.	ICBHI	10 fold
Amaral et al. 2012 [40]	ML	SVM	Acc. = 96.0%, Spec.>94%	Priv.	50 volunteers	N/A
Granero et al. 2018 [9]	ML	DT	Acc. = 87.8%	Priv.	33 patients	N/A
Alghamdi et al. 2024 [3]	DL	CNN	Acc. = 95.1%	Priv.	126 individuals	N/A
Sharma et al. 2023 [43]	DL	CNN	Sens. = 96.59%	Pub.	ICBHI	N/A
Our proposed model	DL	ECAPA	Acc. = 90.86% (Test)	Pub.	ICBHI	Lasso

**Table 11:** Performance metrics with 95% confidence intervals (10-fold cross-validation).

Metric	Mean	Std	95% CI
Accuracy	90.86%	±3.76%	[88.17%, 93.55%]
Precision	96.71%	±2.06%	[95.24%, 98.19%]
Recall	93.65%	±3.30%	[91.30%, 96.01%]
F1-score	95.12%	±2.03%	[93.67%, 96.57%]

## Data Availability

The data that support the findings of this study are publicly available in the Kaggle repository (ICBHI 2017 Respiratory Sound Database) at https://www.kaggle.com/datasets/vbookshelf/respiratory-sound-database (accessed on 24 May 2025).

## Abbreviations

The following abbreviations are used in this manuscript:

TRP	True Positive Rate
FPR	False Positive Rate
ACC	Accuracy
AI	Artificial Intelligence
ANN	Artificial Neural Network
ASP	Attentive Statistics Pooling
CNN	Convolutional Neural Network
COPD	Chronic Obstructive Pulmonary Disease
DL	Deep Learning
DT	Decision Tree
ECAPA	Emphasized Channel Attention, Propagation and Aggregation
ECAPA-TDNN	Emphasized Channel Attention, Propagation and Aggregation Time Delay Neural Network
TDNN	Time Delay Neural Network
SE	Squeeze-and-Excitation
AMP	Automatic Mixed Precision
ICBHI	International Conference on Biomedical and Health Informatics
KNN	K-Nearest Neighbors
MFCCs	Mel-Frequency Cepstral Coefficients
ML	Machine Learning
RNN	Recurrent Neural Network
SHAP	SHapley Additive exPlanations
SVM	Support Vector Machine
XAI	Explainable Artificial Intelligence
